# Steady streaming as a method for drug delivery to the inner ear

**DOI:** 10.1038/s41598-020-79946-z

**Published:** 2021-01-08

**Authors:** Laura Sumner, Jonathan Mestel, Tobias Reichenbach

**Affiliations:** 1 Department of Bioengineering and Centre for Neurotechnology, Imperial College London, South Kensington Campus, London, SW7 2AZ UK; 2 Department of Mathematics, Imperial College London, South Kensington Campus, London, SW7 2AZ UK

**Keywords:** Cochlea, Fluid dynamics, Biomedical engineering

## Abstract

The inner ear, or cochlea, is a fluid-filled organ housing the mechanosensitive hair cells. Sound stimulation is relayed to the hair cells through waves that propagate on the elastic basilar membrane. Sensorineural hearing loss occurs from damage to the hair cells and cannot currently be cured. Although drugs have been proposed to prevent damage or restore functionality to hair cells, a difficulty with such treatments is ensuring adequate drug delivery to the cells. Because the cochlea is encased in the temporal bone, it can only be accessed from its basal end. However, the hair cells that are responsible for detecting speech-frequency sounds reside at the opposite, apical end. In this paper we show that steady streaming can be used to transport drugs along the cochlea. Steady streaming is a nonlinear process that accompanies many fluctuating fluid motions, including the sound-evoked waves in the inner ear. We combine an analytical approximation for the waves in the cochlea with computational fluid dynamic simulations to demonstrate that the combined steady streaming effects of several different frequencies can transport drugs from the base of the cochlea further towards the apex. Our results therefore show that multi-frequency sound stimulation can serve as a non-invasive method to transport drugs efficiently along the cochlea.

## Introduction

Sensorineural hearing loss is caused by damage to the mechanosensitive hair cells in the inner ear, or cochlea, and is one of the most common disabilities across the world^1,2^. Although research into medical treatments such as gene therapy has advanced significantly^3–5^, the progress of effective therapies is stunted by the fact that the inner ear is a very complex part of the body and particularly difficult to access. The cochlea is encased in the temporal bone, the hardest bone in the body. Artificial openings of the temporal bone can disturb the endocochlear potential, an electrical potential across the hair cells that is important for the inner ear’s active process^6,7^. Drug delivery through the cardiovascular system after oral or intravenous application of a drug is also difficult, due to the blood-perilymph barrier segregating the cardiovascular system from the cochlear fluids^8,9^.

The cochlea can be accessed through the membrane-covered oval or the round window at its base that connect to the middle ear. Drugs can be applied either through the middle ear and be deposited near the round window (intratympanic delivery), relying on diffusion through the round window to reach the inner ear, or be injected directly through the round window into the cochlea (intracochlear delivery). In the case of intratympanic delivery, a variety of methods are used in order to maintain contact with the membrane. Hydrogels^10–12^ and gelatin foams^13–16^ can be used as carriers for drugs or micro/nanoparticles in order to extend contact time with the oval or round windows^17,18^, as well as combinations of these in so-called ferrogels which can be manipulated through the use of magnetic fields^19^. Osmotic pumps are also able to extend the delivery timescales^20^. As an alternative invasive approach, a small hole can be drilled into the side of the cochlea and drugs inserted through the opening. However, this so-called cochleostomy alters the functioning of the cochlea and is therefore normally only used for cochlear implants that replace key parts of the inner ear’s function^21^.

In many of these cases, the drugs are delivered at the basal end of the cochlea, where the highest audible frequencies are detected, around 20 kHz in men^6^. However, the frequencies that matter most for speech range from 300 Hz to 3 kHz and are sensed by hair cells further towards the apical end of the cochlea^6,22^. This leads to the next stage of the delivery problem: distribution of the drug once it has been administered to the cochlea. Whilst there are methods such as micropumps^23,24^ which can inject and distribute a drug, most of the current drug delivery methods rely on passive diffusion to reach the mid and apical region. This process is slow, inefficient, and difficult to control or verify^25,26^.

In the work which follows, we assume the drug in question is already present at the basal end of the cochlea, either as a result of intracochlear injection through, or diffusion across, the round window. Once the drug has been administered, active propulsion of drugs along the inner ear can potentially result from steady streaming^27,28^. Steady streaming describes the phenomenon of a net time-averaged fluid motion accompanying an oscillating primary flow field with zero mean^29^. Such a net flow can, for instance, result from Reynolds stresses in boundary layers near no-slip boundaries or from Stokes drift due to differences in the mean motion at a fixed point and the mean motion of a fixed particle moving with the fluid. Both effects result in steady streaming in the cochlea^27,30^.

**Figure 1:**
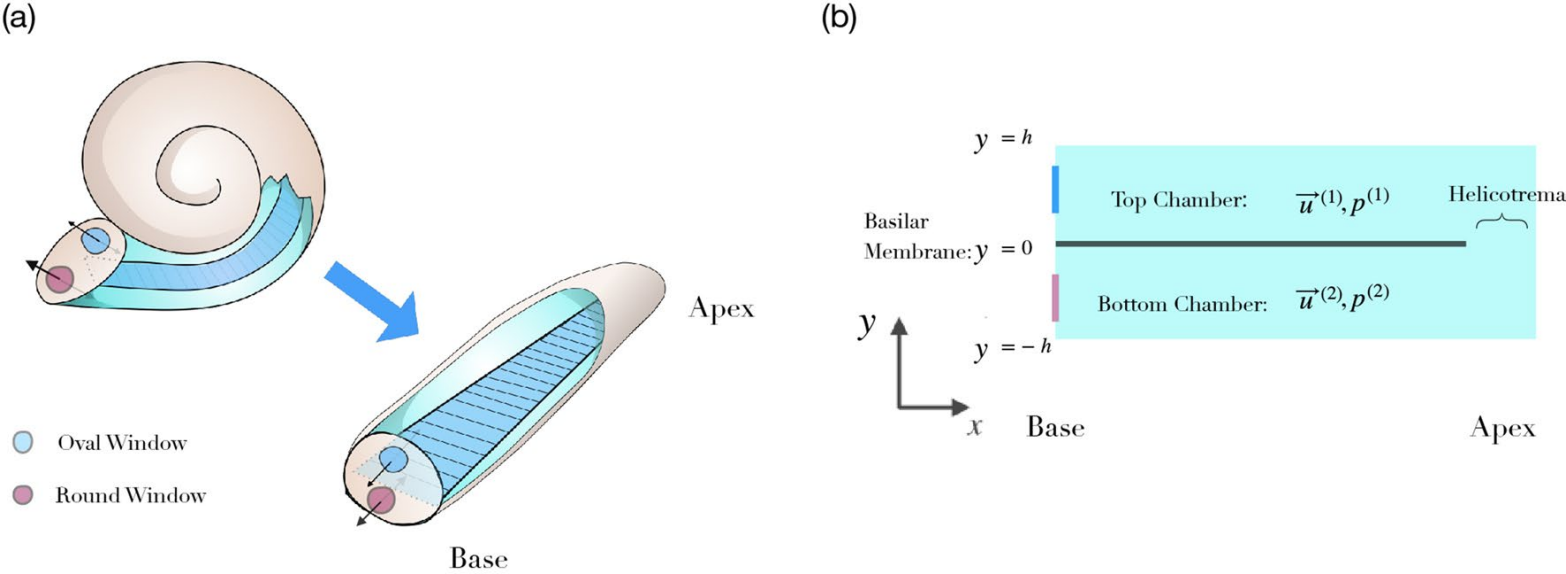
The morphology of the inner ear. (**a**) The cochlea is a spiral-shaped duct filled with fluid and segregated into two chambers by the elastic basilar membrane (dark blue), which can be accessed through the round and the oval windows at the base. Mathematical descriptions of the fluid dynamics typically consider the simplification of an uncoiled, linear cochlea. (**b**) Many aspects of cochlear fluid dynamics can be captured in a two-dimensional representation, in which one dimension, *x*, represents the inner ear’s longitudinal extent and the other dimension, y, denotes the vertical deviation from the basilar membrane. We further denote by $\mathbf {u}^{(1)}$ and $p^{(1)}$ the velocity and the pressure in the upper chamber. Analogously, $\mathbf {u}^{(2)}$ and $p^{(2)}$ are the velocity and the pressure in the lower chamber. Both chambers are connected at the apical end through the so-called helicotrema.

For the purpose of describing the inner ear’s fluid dynamics, the organ can be viewed as consisting of two fluid-filled elongated chambers that are separated by the elastic basilar membrane (Fig. 1). Sound stimulation leads to a displacement of the oval window at the basal end of one chamber, and the induced pressure can be released at the elastic round window at the basal end of the other chamber^22,31^. The sound then leads to a pressure difference across the membrane and therefore to its displacement. The pressure difference and the corresponding membrane displacement travel as waves along the longitudinal extent of the cochlea towards its apex. However, because the impedance of the basilar membrane changes systematically from base to apex, the wave induced by a single frequency exhibits a shortening wavelength and an increasing amplitude as it propagates apically, reaching a peak in amplitude at a characteristic position, beyond which it decays rapidly. This characteristic place is located near the base for high-frequency stimulation and shifts systematically further towards the apex for lower frequencies. The cochlea thereby establishes a tonotopic mapping between frequency and longitudinal location of the wave’s peak. The mapping is approximately logarithmic: frequencies that differ by a constant factor peak at locations that are a constant distance apart^6,22^.

Steady streaming is quadratic in the wave amplitude and so is particularly pronounced near the peak of the wave induced by a pure tone, since the amplitude of the evoked fluid motion there is largest and because of the rapid attenuation beyond the peak location^27,30^. The steady-streaming induced by a single frequency is thus localised in its effect, and can not provide efficient long range drug transport. However, a complex tone, comprising many frequencies could simultaneously excite different regions of the basilar membrane, and particles might be relayed from the vicinity of one excited point to another. In this way, drugs might be transported deep into the inner ear.

Here we combined an analytical approximation of the basilar-membrane wave with computational fluid dynamics simulations and tracking of finite mass particles to investigate the steady streaming induced by a pure tone as well as by complex tones, and to quantify the efficacy of this mechanism for drug transport.

## Methods

### Analytical approximation for the basilar-membrane displacement

We employed an analytical approximation to describe the wave on the basilar membrane. We simplify the spiral geometry of the cochlea by considering a planar membrane with fluid above and below it. The resulting one-dimensional model describes the longitudinal extent of the cochlea through the variable *x* and assumes that the variation in pressure and fluid motion in the vertical dimension is negligible (Fig. 1). The difference *p* in the pressure between the lower and the upper chamber, $p=p^{(2)}-p^{(1)}$, then leads to an upward velocity *V* of the basilar membrane. In a passive cochlea, the relationship between the basilar-membrane velocity and the pressure difference is a linear one, and depends on the frequency of the vibration. For a pure tone of a frequency *f*, and angular frequency $\omega =2\pi f$, the pressure difference can be written as $p=\tilde{p}e^{i\omega t}+\text {c.c.}$ in which “c.c.” denotes the complex conjugate. The resulting displacement of the basilar membrane can similarly be expressed as $V=\widetilde{V}e^{i\omega t}+\text {c.c.}$. We define the complex impedance *Z*(*x*) relating the Fourier coefficients of the pressure and velocity of the basilar-membrane vibration as

1 $$\begin{aligned} \widetilde{V}=\frac{\widetilde{p}}{Z}. \end{aligned}$$

As has been shown previously^31^ the pressure difference then satisfies the wave equation

2 $$\begin{aligned} \frac{d^2 \widetilde{p}}{d x^2} =\frac{2i\omega \rho _0}{Zh} \widetilde{p}, \end{aligned}$$

in which $\rho _0$ denotes the density of the fluid, and *h* is the height of each chamber.

The main difficulty in solving Eq. (2) is the spatial variation of the impedance *Z*(*x*). However, the length scale over which the impedance changes significantly is typically small compared to the wavelength of the resulting wave. As first shown by Lighthill, we can therefore employ the Wentzel-Kramers-Brillouin (WKB) approximation^31,32^. We introduce the complex, *x*-dependent wavenumber

3 $$\begin{aligned} k(x)= \sqrt{\frac{2 \omega \rho _0 }{i Z(x) h}}, \end{aligned}$$

with the square root defined to have a negative imaginary part. The WKB approximation starts from the ansatz

4 $$\begin{aligned} \widetilde{p}(x) = \widehat{p}(x)e^{-i\int _0^x k (x') dx'}, \end{aligned}$$

where the pressure amplitude $\widehat{p}(x)$ is assumed to vary more slowly than 1/*k*, i.e. $k\gg \widehat{p}\,'/ \widehat{p}$. Substituting this expression into Eq. (2) and equating terms of *O*(*k*), requires

5 $$\begin{aligned} \widehat{p}(x)={p_0}\sqrt{{\frac{k_0}{ k(x)}}}=p_0\left( {\frac{Z(x)}{ Z_0}}\right) ^{1/4}, \end{aligned}$$

where $p_0=\widetilde{p}(0)$ is the pressure amplitude applied at the base of the inner ear, $x=0$, while $k_0=k(0)$ and $Z_0=Z(0)$. The velocity of the basilar membrane follows as

6 $$\begin{aligned} V(x,t)=V_0\left( {\frac{Z_0}{Z(x)}}\right) ^{3/4}e^{-i\int _0^x k (x') dx'+i\omega t} + \text {c.c.} \end{aligned}$$

where $V_0=p_0/Z_0$ is the velocity amplitude at the basal end. It is possible to further analyse the WKB integral asymptotically, and obtain a formula in terms of Bessel functions (see for example^33^), but we used Eq. (6) directly in the numerics below.

### Parameter values

We model the impedance of the basilar membrane in terms of its stiffness *K*, mass *m*, and damping $\xi$ per unit area. All of these can depend on the longitudinal location *x* and we allow for nonlinear effects by permitting $\xi$ to vary with amplitude

7 $$\begin{aligned} Z(x)=[i\omega m(x) + \xi (x,\,p_0) - iK(x)/\omega ]/A. \end{aligned}$$

The cross-sectional area *A* represents a transverse strip of the basilar membrane with the width of one hair cell, 8 $\upmu \hbox {m}$, and a breadth of 186 $\upmu \hbox {m}$^31^. Stiffness, mass and damping refer henceforth to this narrow section of the basilar membrane.

In the absence of damping, at each frequency there is a position of resonant response, where $\omega ^2=K/m$. The small damping coefficient ensures the regularity of the solution, but maintains the link between frequency and cochlear position. The damping derives from two sources; firstly, the viscous damping of the fluid, which is linear at low Reynolds number, and secondly, the nonlinear cochleal response as described below.

Experimental data regarding the different contributions to the basilar-membrane impedance are not reliably available in humans. We therefore based our computations on experimental data from the gerbil’s inner ear. The hearing range of gerbils, about 200 Hz to 60 kHz, overlaps largely with that of humans, which extends from 20 Hz to 20 kHz. Such data show that the stiffness of the basilar membrane decays exponentially from base to apex, at least between the basal and the mid region of the inner ear^34^. The main contribution to the basilar-membrane mass *m*(*x*) derives from the organ of Corti on the membrane. Since the cross-section of the organ increases from base to apex, the basilar-membrane mass also increases^35^.

We assume exponential dependencies of both stiffness and mass on the longitudinal location, corresponding to the cochlea’s logarithmic tonotopic mapping between frequency and spatial location: $m= m_0 e^{x/l_m}$ and $K= K_0 e^{-x/l_K}$. The parameters $m_0$ and $K_0$ denote the mass and stiffness at the basal end, $x=0$, and $l_m$ and $l_K$ are the length scales that correspond to the exponential variation. We obtained these parameters from experimental measurements in the gerbil cochlea. The cross-sectional area of the organ of Corti, which sits on the basilar membrane, at the basal end is about 8000 $\upmu \hbox {m}^2$, implying a moving mass of about $m_0=32$ ng^31,35^. The stiffness of the membrane at this location is about $K_0=1$ N/m^34^. At a location of 7 mm apical from the basal end, the cross-sectional area of the organ of Corti has increased to about 18,000 $\upmu \hbox {m}^2$, yielding a moving mass of the basilar membrane of about 65 ng. The stiffness of the membrane at this cochlear location has decreased to 0.03 N/m. These changes in stiffness and mass imply the length scales $l_m=115$ nm and $l_K=501$ nm. For the height of each fluid filled chamber in the gerbil’s inner ear we have assumed $h=0.5\,\hbox {mm}$.

**Figure 2:**
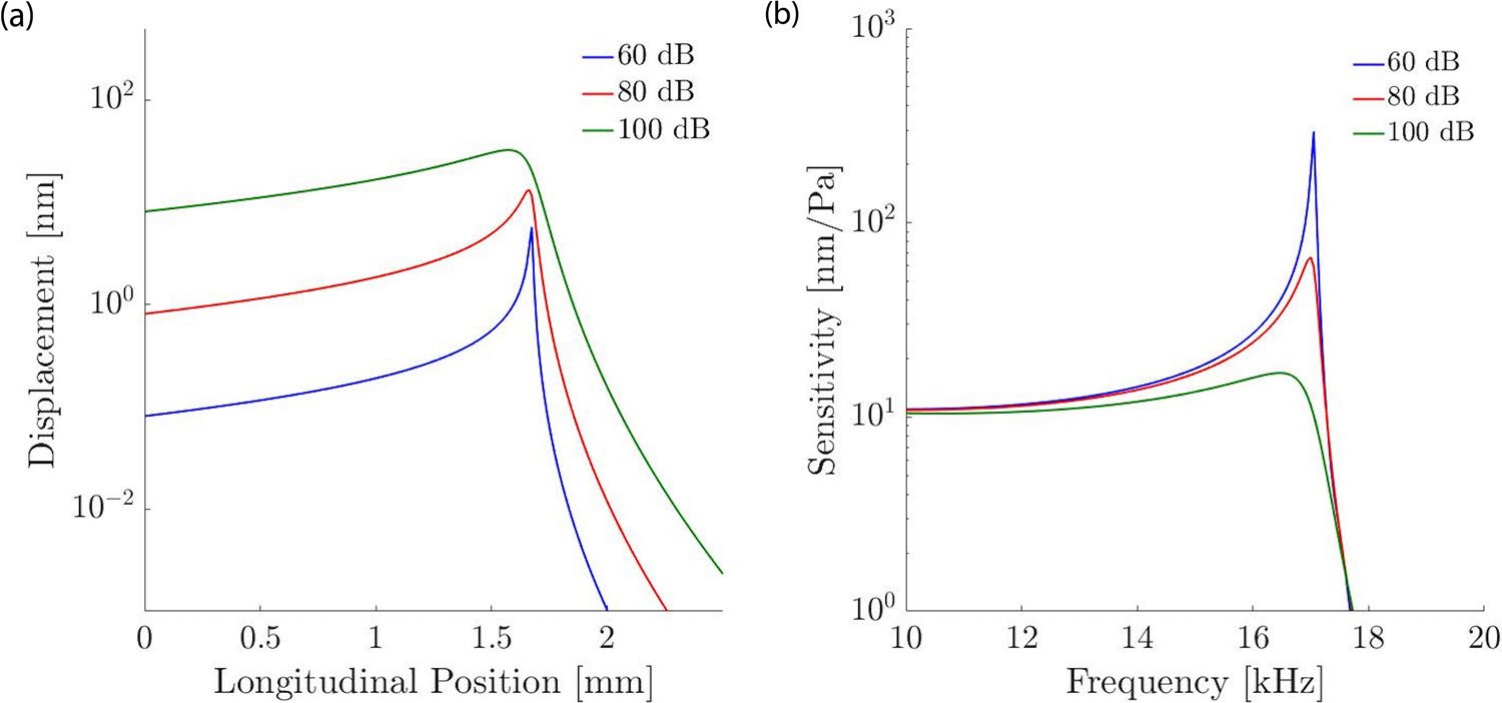
Amplitude dependent response of the basilar-membrane near the peak, modelled using a level-dependent drag coefficient $\xi$ in the membrane impedance. (**a**) The membrane displacement increases linearly with the stimulation intensity basal and apical to the peak, but sublinearly at and near the peak. (**b**) The nonlinear response emerges clearly in the sensitivity, defined as the ratio of the membrane’s vibrational amplitude to the applied sound pressure. Where the sensitivity is independent of the sound-pressure level the membrane response is linear, but nonlinear otherwise. The compressive nonlinearity near the wave’s peak shows that the peak is sharper at lower stimulation intensities.

At each longitudinal location *x*, mass and stiffness define a resonance frequency $f_0=\sqrt{K/m}/(2\pi )$ at which the contributions to the basilar membrane impedance from stiffness and mass cancel. The wave induced by stimulation at frequency *f* peaks at that location, with an amplitude that is limited by the drag portion of the basilar membrane impedance. A healthy inner ear has, however, an active process that provides mechanical amplification of weak sound vibrations, effectively counteracting the viscous drag^22,36,37^. This amplification varies with sound-pressure level: small vibrations are amplified most, and larger vibrations successively less. This leads to a compressively nonlinear response of the basilar-membrane vibration that can, for instance, be explained through the membrane’s segment being poised near a Hopf bifurcation^38–40^. As another consequence, the peak of the basilar-membrane wave is sharper at low rather than high sound pressure levels.

Here, we modelled this level-dependent amplification through considering a spatially constant, amplitude dependent damping $\xi (p_0)$. In particular, we considered three distinct sound-pressure levels, and adjusted the damping parameter $\xi$ so that the peak of the basilar-membrane displacement as predicted by the WKB approximation (6) had a width corresponding to experimental data^41^. We performed the analysis for a stimulation frequency of 17 kHz and the three sound-pressure levels of 60, 80 and 100 dB SPL (Fig. 2). For these sound levels we identified respectively the coefficients $\xi =10$ nNs/m, $\xi =500$ nNs/m, and $\xi =2$ $\upmu \,$Ns/m for the corresponding total drag, including viscous and amplification effects. These drag coefficients reproduced both the experimentally-observed nonlinear response of the basilar-membrane vibration at the peak and the broadening of the peak for larger sound intensities.

### Computational fluid dynamics (CFD)

Computational fluid dynamic simulations were carried out in OpenFOAM v 3.0.1., a finite volume, open source computational fluid dynamics toolkit. We simulated the fluid flow in a two-dimensional model of the cochlea, in which two parallel chambers were separated by the basilar membrane but were connected at the apical end (Fig. 1). Using the known solution for the basilar membrane waves, the time-dependent displacement of the membrane was obtained from the numerical solution of the WKB approximation, Eq. (6), using MATLAB. The output of the MATLAB analysis was a matrix whose rows corresponded to the set of membrane coordinates at a given time step. This was then used as an input to the fluid simulation, allowing the membrane’s boundary to be updated at each time step and the fluid to react accordingly. OpenFOAM’s built in dynamic mesh capabilities were used to ensure mesh conformation. No-slip boundary conditions were imposed at the membrane and all boundaries. Through this approach, the fluid-structure interaction between the cochlear fluids and the basilar membrane was reduced to a purely fluid-dynamic problem.

Fluid flow in the inner ear results from motion of the middle ear and resulting movement of the oval and round windows. However, because our approach utilised an analytically-derived basilar-membrane motion, we did not need to model the movement of the round and oval window. We therefore did not need to solve the fluid-structure-interaction of the fluid with the different membranes of the inner ear, but could focus on the fluid dynamics only.

The Reynolds number of the flow is low, although the Womersley number need not be, so that the Navier-Stokes equations reduce to the unsteady Stokes equations. To leading order, therefore, the fluid velocity consists of a single harmonic, $e^{i\omega t}$. However, second order interactions give rise to a steady-streaming velocity, with a steady component which can lead to net transport down the cochlea. The domain is two-dimensional, though at high Womersley number, derivatives normal to the plate dominate, and an analytic solution can be written down. However, even in that limit the particle paths need to be found numerically, and so we preferred to use computational simulation from the start, for all parameter values.

We adapted the solver BMicoFoam that was created through the combination of the icoFoam and solidParticle solvers. The icoFoam solver is a transient incompressible flow solver, which uses an implicit Euler method for time integration, and a discrete volume Gauss linear scheme for the spatial differentials. The fluid flows were visualized through paraView. Post-processing was completed separately by analysing the OpenFOAM output in MATLAB.

**Figure 3:**
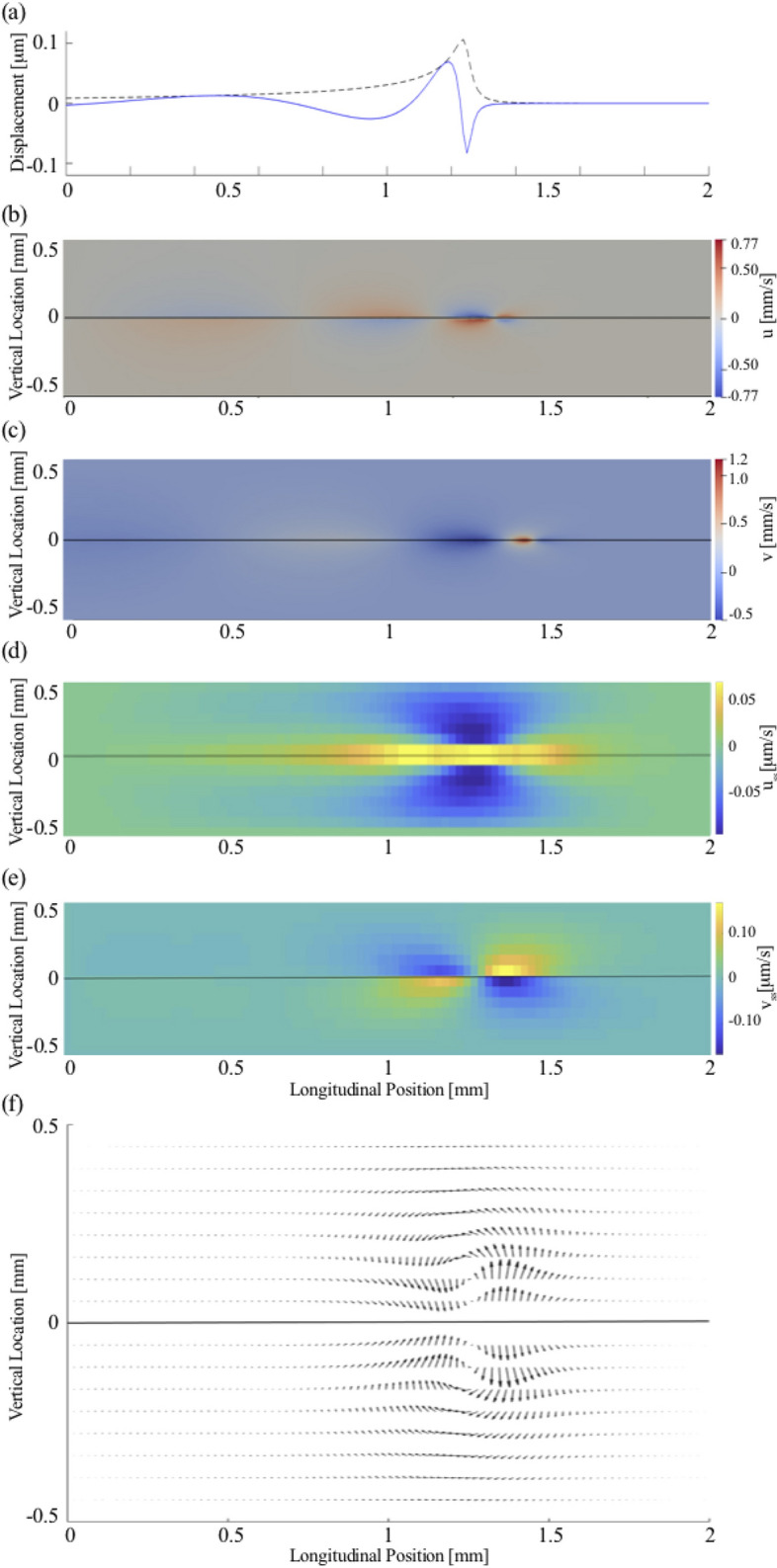
Steady streaming induced by a pure tone of 20 kHz at 80 dB SPL. (**a**) The wave on the basilar membrane (blue line) has a wavelength that shortens upon approaching the resonant position. The wave amplitude (black dashed line) peaks at the resonant position. OpenFOAM results for the instantaneous longitudinal (**b**) and vertical (**c**) fluid field velocity components, (*u*, *v*) which are both largest near the resonant position. (**d**) The longitudinal component of the steady streaming, $u_{ss}$, is largest near the peak of the basilar-membrane wave as well. Near the membrane, the velocity is oriented towards the apex, and further away from the membrane particles move towards the base. (**e**) The vertical streaming velocity, $v_{ss}$ points towards the membrane basal to the wave’s peak, and away from the membrane apical to the peak. (**f**) The steady streaming flow field shows two counter-rotating vortices, one in the upper and one in the lower chamber.

The timestep, $\Delta t$ was determined by the stimulation frequencies. For multi-frequency forcing, in order to resolve the particle trajectories well, we employed ten timesteps per cycle of the highest frequency used in the stimulation. For single-frequency forcing a smaller timestep can be used for the same computational cost. The spatial discretisation used a triangular Delaunay mesh. The mesh-size $\Delta x$ was chosen to be small enough to capture the basilar-membrane motion accurately, and also to resolve the thickness of the Stokes boundary layer at higher Womersley numbers. The Courant number, $C_0= (u_{max} \Delta t)/\Delta x$, in which $u_{max}$ is the largest velocity present in the flow was kept much less than 1 to obtain stable particle trajectories. Accordingly, the mesh size varied from $22.5\,\upmu$m near the cochlear boundaries to $11.2\,\upmu$m at the basilar membrane to provide increased spatial resolution near the membrane.

The cochlea is filled with perilymph and endolymph, specialized ionic solutions whose density and viscosity are similar to that of water. We therefore considered a density of $\rho _0=1000\,\hbox {kg}\,\hbox {m}^{-3}$ and a kinematic viscosity of the fluid of $\nu = 10^{-6}\,\hbox {m}^2\,\hbox {s}^{-1}$.

For particle tracking, 5000 neutrally buoyant particles with an almost elastic collision model (coefficient of restitution of 0.95), a diameter of 200 nm^17^ and a friction coefficient of $10^{-3}$ were injected at zero velocity into the basal region of the two chambers. Note that these particles have an associated mass and are not passive point particles as have been considered by other authors^27,42^. In this way, the work accounts not only for passive particles in solution, but also for low-solubility particles in suspension as used in certain therapeutical approaches^26,43^. Particles were evenly spaced on a grid covering most of the basal region of each chamber. Their trajectories were calculated throughout the simulation. The components of the steady streaming velocity, $(u_{ss}, v_{ss})$ were then extracted from the particle trajectories through computing the temporal averages of the velocities.

**Figure 4:**
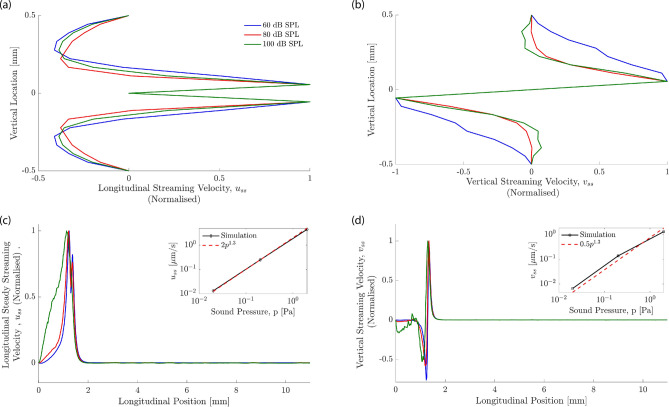
Steady-streaming velocity profiles at different sound intensities for 20 kHz stimulation taken at $x=1.2\,\hbox {mm}$, the 20 kHz resonance location. The velocity profiles have been normalized by the maximal velocity. (**a**) The longitudinal streaming velocity is largest close to the basilar membrane. The vertical extent around the basilar membrane over which fluid flow occurs towards the apex is slightly larger at higher sound intensities. (**b**) The vertical streaming velocity peaks at a similar distance from the basilar membrane as the longitudinal component. The kinks seen in these profiles are an artefact of the coarse resolution of the streaming field but do not affect the overall accuracy of the calculation. (**c**) The longitudinal streaming velocity close to the basilar membrane. The inset shows that the maximal value of $u_{ss}$ increases approximately as a power law with the the sound pressure *p*: $u_{ss}\sim p^{1.3}$. (**d**) The vertical streaming velocity shows that, for high frequencies at higher sound-pressure levels (eg. 100 dB SPL), the boundary at the base of the cochlea influences the flow. The inset shows the power law dependence of the maximal value of $v_{ss}$ on the sound pressure level *p* which follows approximately a power law, $v_{ss}\sim p^{1.3}$.

Steady streaming is a nonlinear phenomenon, and its amplitude is proportional to the square of the fluid velocity^27,30,44,45^. Small stimulation intensities therefore lead to only tiny steady streaming velocities that require long computational times to measure. However, while the oscillatory motion must be resolved on the oscillatory time-scale, the steady component does not. For computational efficacy, and for the multiple-frequency stimulation presented below, we therefore scaled both the basilar membrane motion, as well as the fluid velocities at 80 dB SPL, by a factor of 300. We then computed the resulting fluid flows and steady streaming, and scaled them back by the factor of 300 as well as by its square, respectively. This method resulted in a significant reduction of the required computation time without affecting the obtained results.

## Results

### Pure-tone stimulation

We first investigated the steady streaming caused by stimulation with a pure tone. In agreement with analytical descriptions as well as computational results, we found that pure-tone stimulation elicits oscillatory fluid flow in both cochlear chambers, with an amplitude that is largest near the peak of the basilar-membrane wave (Fig. 3).

The particle tracking that we employed showed the accompanying steady streaming, in particular the Lagrangian streaming field (Fig. 3). We observed the formation of a pair of counter-rotating vortices above and below the basilar membrane, at the location of the wave’s peak. We then investigated the influence of the stimulation intensity on the shape of these vortices. We found only a minor change, with the vortices induced by a higher intensity being slightly wider in the vertical direction (Fig. 4). The steady streaming velocity depends on the sound pressure level *p* approximately through a power law with a power of 1.3: $u_{ss}\sim p^{1.3}$ and $v_{ss}\sim p^{1.3}$. This is less than the quadratic increase with pressure expected from a passive cochlea with a linear response, and reflects the active’s cochlear compressive nonlinearity that we have modelled through a level-dependent damping parameter (Fig. 2).

### Multi-frequency stimulation

**Figure 5:**
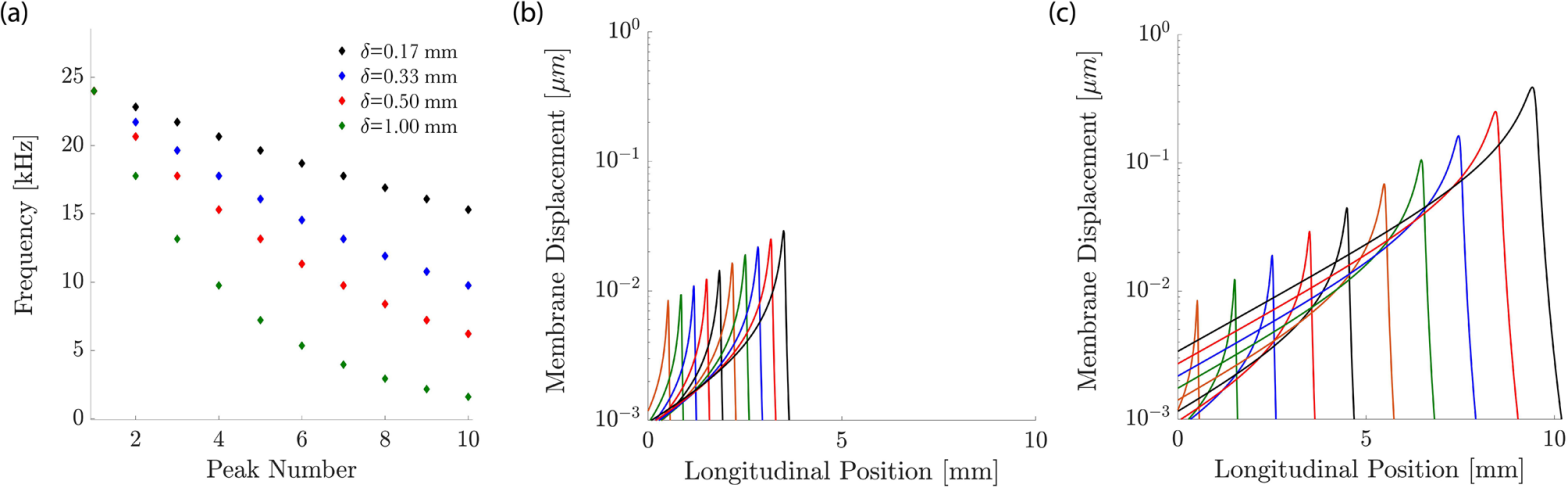
Multi-frequency stimulation. (**a**) We choose discrete frequencies so that successive frequencies differ by a constant ratio, leading to a constant spacing $\delta$ of the peaks of the different induced basilar-membrane waves. (**b**) When the frequencies are close such that the spacing between the peaks of the induced waves is small, $\delta = 330\,\upmu \hbox {m}$, we obtain significant overlap between the amplitudes of the individual waves. (**c**) A larger spacing between the successive peaks, $\delta = 1\,\hbox {mm}$, leads to less overlap.

Because pure-tone stimulation elicited significant steady streaming only near the peak of the basilar-membrane wave, we investigated whether a complex tone with several frequencies could lead to steady streaming within a larger longitudinal range within the cochlea (Fig. 5). We therefore considered frequencies $f_1$, $f_2$, ..., $f_n$ that differed by a constant factor $R>1$: $f_{i+1}=Rf_i$ for $i=1$, 2, ..., $n-1$. Due to the cochlea’s logarithmic tonotopic map, the locations $x_1$, $x_2$, ..., $x_n$ at which the basilar-membrane waves elicited by stimulation at frequency $f_1$, $f_2$, ..., $f_n$ peaked decreased linearly with the logarithm of the stimulation frequency: $x_i=-m\ln (f_i)$. The spatial distance $\delta$ between two neighbouring peak locations $x_i$ and $x_{i+1}$ was therefore constant: $x_{i}-x_{i+1}=\delta$ for $i=1$, 2, ..., $n-1$. The spatial constant $\delta$ was proportional to the logarithm of the frequency ratio *R*, $\delta =m\ln (R)$, with a proportionality constant $m=3.3\,\hbox {mm}$. In our simulations we explored how the spatial spacing $\delta$ affected the resulting steady streaming.

Two factors limited the spacings that we could meaningfully consider. First, our simulations of pure-tone stimulation revealed that the spatial extent of the induced steady streaming was on the order of 1 mm. Spacings of subsequent wave peaks of more than 1 mm will therefore not lead to overlapping vortices, and thus not to a combined steady streaming. Second, the region around the peak of the wave induced by a pure tone in which the basilar-membrane response is nonlinear is at least 100 $\upmu \hbox {m}$ long^46^. Although we accounted for the nonlinear response through a level-dependent drag coefficient in the basilar-membrane impedance, our analytical approximation did not capture further nonlinear effects. In particular, the approximation we employed did not account for the two-tone suppression that results when the basilar-membrane waves induced by two nearby frequencies overlap in their region of nonlinear response^47–49^. We could therefore not reliably simulate spacings of 100 $\upmu \hbox {m}$ or less.

**Figure 6:**
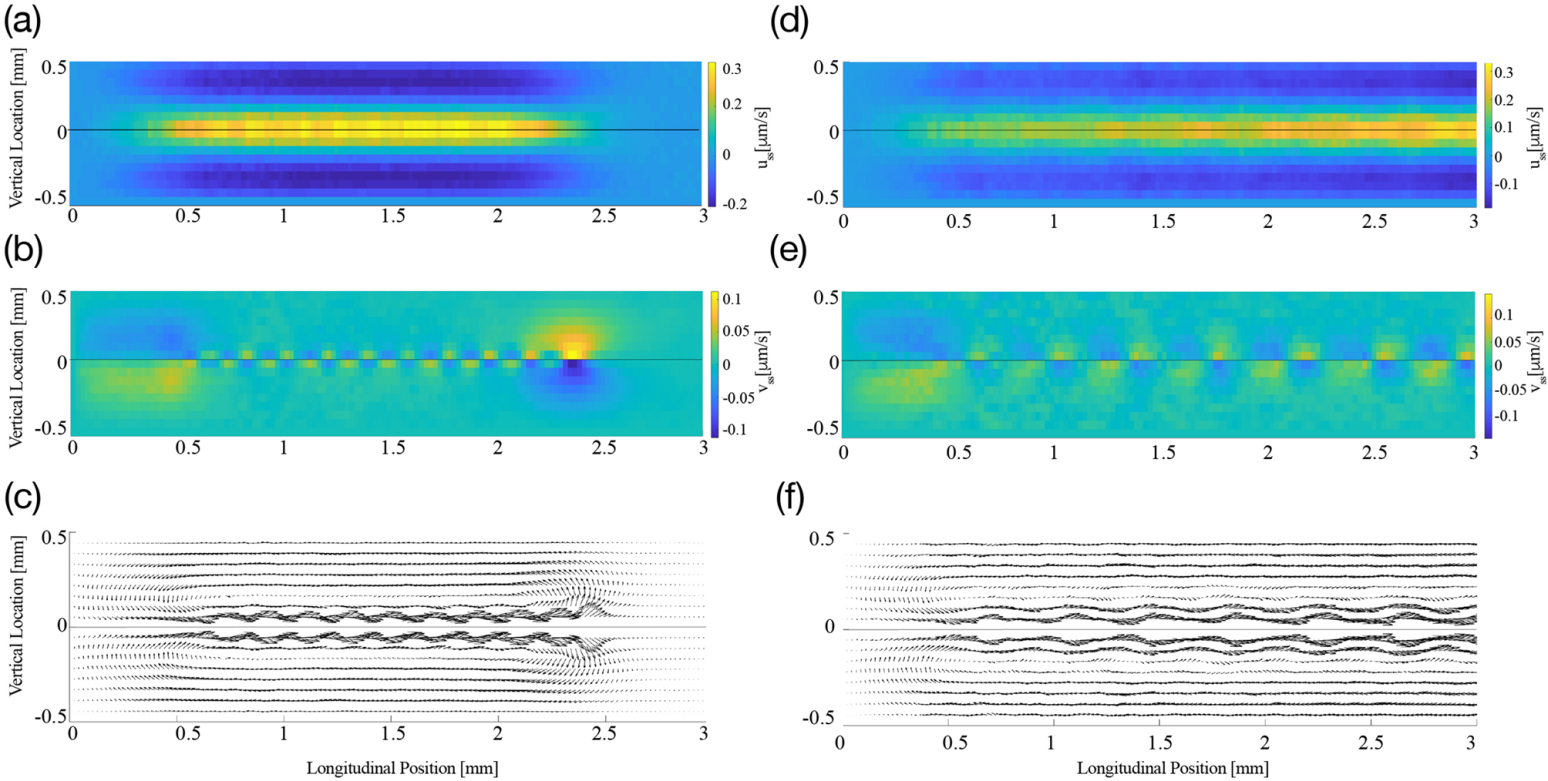
Steady streaming induced by multi-frequency stimulation at 80 dB SPL. (**a**–**c**), The velocities of steady streaming that results from stimulation at frequencies whose individual basilar-membrane waves peak at a spatial distance of $\delta =170\,\upmu \hbox {m}$ apart from one another. (**a**) The longitudinal fluid flow has a significant amplitude in the extended region from 0 to 2.5 mm, where the waves induced by the individual waves peak. (**b**) The vertical velocity is strongest at the beginning and at the end of that region, that is, near the cochlear base as well as about 2.5 mm apical to it. (**c**) The vector fields show how the steady-streaming vortices associated with each individual frequency combine to a large circular flow that extends significantly along the cochlea. (**d**–**f**) The steady streaming velocity induced by a multi-frequency stimulation in which the individual peaks of the induced basilar-membrane waves are further apart, $\delta =330\,\upmu \hbox {m}$. Significant steady streaming emerges along a longer longitudinal range of the cochlea, because the multi-frequency stimulation extends to lower frequencies. Because the wave peaks are further apart, the steady-streaming vortices associated to the individual frequencies emerge stronger than for the case of closer frequencies.

**Figure 7:**
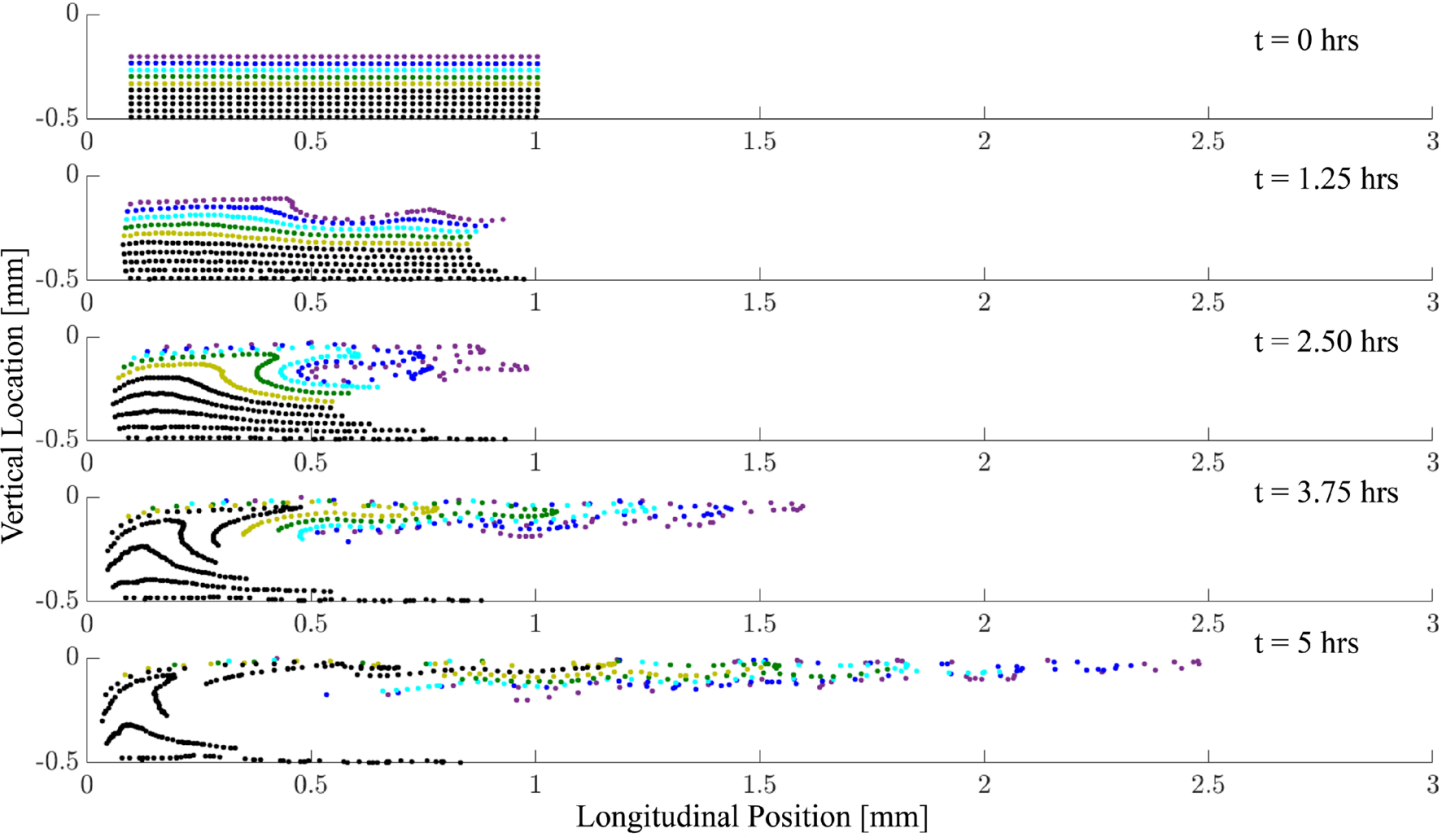
Individual particles near the cochlear base (coloured dots) are displaced throughout the cochlea due to steady streaming. We show the particle displacement in the bottom channel as induced by multi-frequency stimulation at 80 dB SPL with a frequency separation of $\delta =330\,\upmu \hbox {m}$. Particles near the membrane (purple) are quickly displaced towards the base, while particles away from the membrane (black) are first moved towards the membrane and then towards the apex.

**Figure 8:**
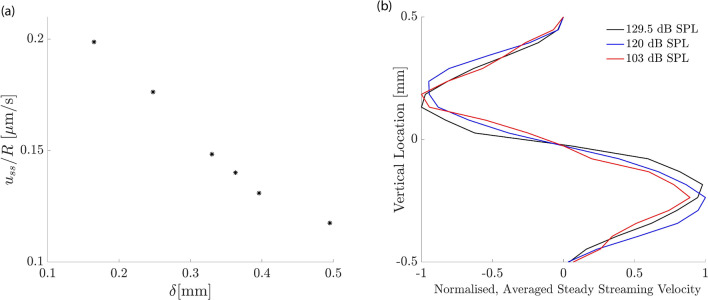
Influence of sound intensity. (**a**) The longitudinal component of the average steady streaming, $\overline{u}_{ss}$ at the base of the cochlea, normalised by the ratio of the successive frequencies in the multi-frequency stimulation *R*. If the steady streaming were proportional to the energy dissipated along a constant cochlear extent, then this normalized velocity would be constant. However, the normalized velocity increases strongly for smaller spatial separations, evidencing that the steady streaming becomes stronger when the multi-frequency stimulation uses many pure tones that successively differ only by a small amount. (**b**) Vertical profile of the vertical average steady-streaming velocity 0.1 mm from the base of the cochlea, the basal edge of the circulation region for $\delta = 170\,\upmu \hbox {m}$, as shown in Fig. 6 and its dependence on the sound intensity. The stimulation intensity has only little influence on the velocity profile. The velocity profiles have been normalized by the maximal value at each sound pressure level.

Our numerical results showed that such multi-frequency stimulation leads indeed to significant steady streaming along a considerable extent of the inner ear (Fig. 6). This effect emerged because the steady-streaming vortices induced by the individual frequencies combined to create an effective “streaming channel” along which the particles are transported longitudinally. Figure 7 details the positions of the injected particles at different points in time throughout the simulation in the bottom half of the channel. It shows how the particles are pulled into a streaming channel near the basilar membrane that is formed by the combined eddy structure. Our results also evidenced that a smaller separation between frequencies led to stronger longitudinal steady streaming (Fig. 8). Moreover, our CFD simulations show significant vertical velocity of the steady streaming near the base of the cochlea. This vertical velocity was, in either chamber, directed towards the basilar membrane, and implied that particles near the base were transported near the membrane and then propelled longitudinally along it.

We also considered whether particles injected at the base but far from the basilar membrane would be transported to the membrane as a result of the steady streaming. We found that this was indeed the case: the steady streaming near the base has a significant vertical component that is directed towards the basilar membrane (Fig. 6). Due to the nonlinear basilar-membrane response to sound intensity, which we modelled through an intensity-dependent drag coefficient of the basilar membrane, the spatial profiles of the steady streaming could, in principle, depend on the sound intensity. We confirmed, however, that the vertical velocity profile depended only little on the intensity level of the sound, up to an overall scaling (Fig. 8b). This result agrees with our finding that the intensity of a pure tone has little influence on the spatial velocity profile as well (Fig. 4), and confirms that particles injected at the base are transported effectively towards the membrane, independent of the sound intensity.

## Discussion

This work investigated the effect of different sound stimuli on the fluid mechanics of the inner ear. Although computational simulation of both the fluid mechanics^50,51^ as well as the steady streaming^27,52^ and drug distribution^25^ have been reported, tracking of finite mass particles (i.e. not passive) individual particles in the flow has not. Here we validated our model against Lighthill’s analytical work on the subject and subsequently used the model to investigate the most effective way to harness the streaming. The aim of the work was to determine whether there is a combination of tones which could deliver (or at least better redistribute) a drug which has either diffused across the round window or been injected through it directly.

Each pure tone resulted in an eddy in each cochlear chamber whose apical edge caused particle translation back towards the base of the cochlea. Playing a single tone therefore serves to rotate the particles locally in the region of the characteristic place. By inducing another eddy apical to the first, the backward drift of particles can be avoided: instead they are “pulled” into the flow of the proceeding eddy. Because the eddy is at a lower frequency at the same sound-pressure level, the amplitude is higher and so therefore is the velocity, leading to an acceleration along the channel. By continuing to increase the number of eddies, this “streaming channel” along which particles are accelerated is extended and hence the region over which the drug is distributed increases. By choosing the correct frequency combination, the effect can be optimised by reducing the vertical component of particle velocity. This can be seen in Fig. 6 where panels (a)–(c) show that the closer eddy combination results in a diminished vertical steady streaming velocity inside the global eddy compared to the edge. Importantly, as presented in Fig. 8, although the eddy strength is reduced with sound-pressure level, the penetration depth and hence the region of influence of the eddies does not change. These results therefore show that a multiple frequency stimulus not only generates larger velocities in the cochlea by creating a streaming channel along which particles are accelerated, but also does not require a specific injection location at the base in order to be effective.

Starting from the proof-of-concept undertaken here, further work will be required to develop the method towards clinical application. In particular, it will be important to use experimental data for the human cochlea rather than for the rodent cochlea that we investigated here. It will also be beneficial to investigate a more complex, three-dimensional and realistic geometry of the cochlea, as well as to include the interaction of the fluid with the different membranes, to determine the dependence of the streaming on factors such as boundary effects. In particular, including the motion of the basilar membrane in a fluid-structure interaction simulation will allow one to model the longitudinal motion of the basilar membrane, which has been proposed by Edom et al.^27^ to increase the streaming velocity. Finally, including the active process will allow to model the width of the basilar-membrane waves induced by a pure tone and their dependence on the sound-pressure level. This will be necessary to optimise the sound stimuli for clinical application, in particular regarding the choice of the frequency separation parameter $\delta$ that one can use in order to create the streaming channel.

Sensorineural hearing loss therapies are lacking an effective mode of delivery of the pharmaceuticals to the damage site. The purpose of this work was to investigate whether or not the phenomenon of steady streaming, which is already present in the inner ear, could be a viable means of overcoming this hurdle. From the results presented here, we believe that it shows promise. Specifically, we find that a superposition of optimally separated frequencies results in the formation of a “streaming channel”, capable of transporting a drug from a broad region near the base of the cochlea a predetermined distance along it. In all simulations presented here, 10 frequencies were chosen to be superposed. By increasing or decreasing this value, for a constant $\delta$, the longitudinal reach of the streaming channel will either increase or decrease. In this way, we can achieve targeted delivery relatively simply.

We also show that the timescales over which these stimuli must be played to achieve this transport are reasonable for therapies. From Fig. 6d, considering a particle initially injected at 0.25 mm, the time taken to reach 3 mm (approx 1/3 of the length of the cochlea) by accelerating from 1 $\upmu \hbox {m/s}$ to 3 $\upmu \hbox {m/s}$ is around 5 h. This timescale is well within the recommended exposure limit suggested by both the OSHA (16 h at 80 dB SPL) as well as the NIOSH (25 h at 80 dB SPL) in the CDC guidelines^53,54^.

Previous work by Edom et al.^27^ has estimated the steady streaming velocity for a pure tone at 1 kHz and 94 dB SPL to be about 300 $\upmu \hbox {m/s}$. This result is in approximate agreement with the steady streaming velocities that we reported here, such as our finding that a 20 kHz tone presented at 80 dB SPL leads to a steady streaming velocity of about 300 nm/s. The difference in stimulation intensity of 14 dB SPL between our computation and the previous one indeed leads to a factor of 5 in the stimulation amplitude, and hence to a factor of 25 in the resulting steady streaming. This leaves a difference of a factor of 40 between the steady streaming velocity reported by Edom et al.^27^ and the one that we have computed. This factor presumably results from the different frequencies that were considered: lower frequencies are indeed known to lead to larger basilar-membrane displacement than higher frequencies, at the peak of the traveling wave. We note that, since the steady-streaming velocity increases in proportion to the square of the basilar-membrane velocity, a factor of about 7 in the basilar-membrane displacement at the different frequencies and characteristic locations already suffices to explain the additional factor of 40 between the obtained steady-streaming velocities.

Although the streaming velocities are admittedly small, over long enough therapy times and with optimised sound stimuli, there is potential for steady streaming to become a way to non-invasively deliver a drug once administered to the cochlea. Now that we have demonstrated a proof of concept for non-invasive drug delivery in the passive cochlea, there is a rich landscape of sound stimuli to explore.
